# Unsupervised 20-feature immunohistochemical immune profiling identifies exploratory cervical cancer phenotypes with unfavorable survival signals

**DOI:** 10.3389/fonc.2026.1914836

**Published:** 2026-08-24

**Authors:** Angel Danchev Yordanov, Eva Tsoneva, Polina Damqnova Dimitrova, Stoyan Georgiev Kostov, Ihsan Hasan, Velizar Stefanov Shivarov

**Affiliations:** 1 Department of Gynecologic Oncology, Medical University Pleven, Pleven, Bulgaria; 2 Clinic of General and Oncogynaecology, Military Medical Academy, Sofia, Bulgaria; 3 Department of General and Clinical Pathology, Heart and Brain Center of Clinical Excellence, Pleven, Bulgaria; 4 Department of Gynecology, Hospital “Saint Anna”, Medical University “Prof. Dr. Paraskev Stoyanov”, Varna, Bulgaria; 5 Research Institute, Medical University–Pleven, Pleven, Bulgaria; 6 Obstetrics and Gynaecology Center “ Tiara”, Sofia, Bulgaria; 7 Department of Clinical Hematology, St. Sophia General Hospital, Sofia, Bulgaria

**Keywords:** cervical cancer, competing risk, exploratory biomarker, hierarchical clustering, immunohistochemistry, tumor immune microenvironment

## Abstract

**Objective:**

To determine whether unsupervised integration of routinely accessible immunohistochemical immune features can identify clinically relevant tumor immune microenvironment phenotypes in cervical cancer.

**Methods:**

We performed a retrospective exploratory analysis of surgically treated cervical cancer cases with complete scoring for a 20-feature immunohistochemical immune panel. The features were derived from approximately 11 antibody-defined markers, including separate intratumoral (IT) and stromal (ST) scores where applicable. Hierarchical clustering was performed using standardized feature values, Euclidean distance, and Ward.D2 linkage. The three-cluster solution was the primary unsupervised analysis. The comparison between C2 and combined C1/C3 was an exploratory, outcome-informed contrast generated after inspection of the primary three-cluster survival curves. Cluster-defining features, clinicopathological associations, overall survival, cause-specific Cox models, and competing-risk endpoints were assessed.

**Results:**

The source dataset included 226 patients; 177 had pT1b-region disease and 169 had complete data for the 20-feature panel. Three tumor immune microenvironment clusters were identified: C1 (n=50), C2 (n=78), and C3 (n=41). The most discriminatory features were CD8-ST, CD8-IT, CD3-ST, PD-1-ST, PD-1-IT, CD3-IT, CD4-ST, CD68-IT, CD20-ST, and CD57-ST. C2 showed relative depletion of several stromal and intratumoral lymphoid and checkpoint-associated features. In the exploratory C2 versus C1/C3 contrast, C2 was associated with inferior overall survival by Kaplan-Meier analysis (log-rank p=0.012) and univariable Cox regression (HR 2.14, 95% CI 1.16-3.92, p=0.014). After adjustment for age, nodal status, and histology, the association was attenuated and borderline (HR 1.84, 95% CI 0.99-3.42, p=0.055). Fine-Gray analysis showed a similar adverse direction for neoplasm-specific death (SHR 2.37, 95% CI 0.95-5.87, p=0.063).

**Conclusion:**

Routine compartment-aware immunohistochemical immune profiling identified exploratory cervical cancer immune phenotypes, including an immune-depleted C2 cluster with unfavorable survival signals. These findings are hypothesis-generating and require independent validation before clinical implementation.

## Introduction

Cervical cancer remains a clinically heterogeneous malignancy despite established clinicopathological staging systems and advances in systemic therapy (1–4). Immune checkpoint inhibition has strengthened the rationale for characterizing the cervical cancer tumor immune microenvironment, but clinically usable immune stratification remains incompletely defined (4, 5).

Tumor-infiltrating lymphocytes, regulatory T-cell populations, macrophages, B-cell aggregates, natural killer cell-related markers, and checkpoint molecules have each been investigated in cervical cancer and other solid tumors (6–13). These studies support the biological importance of the immune microenvironment but also highlight a recurring problem: single-marker analyses can be inconsistent because they ignore coordinated immune programs and the spatial distinction between tumor nests and surrounding stroma (14–16).

Our previous work in Bulgarian cervical cancer cohorts examined CD47, CD8, CD68, CD4, FOXP3, and other immune or immunomodulatory markers individually or in smaller marker constellations (17–20). The present study extends that work by integrating a broader routine immunohistochemical feature panel while retaining compartmental assessment of intratumoral and stromal signals. We aimed to identify exploratory tumor immune microenvironment phenotypes, define the between-cluster feature differences that distinguish them, and test whether these phenotypes show unfavorable overall and cause-specific survival signals.

## Methods

### Study design and cohort

This retrospective cohort study used archived formalin-fixed paraffin-embedded cervical cancer tissue and linked clinicopathological data from patients treated at the Department of Oncological Gynaecology of the Georgi Stransky University Hospital, Pleven, between 2014 and 2023. The source dataset contained 226 patients. The analytic cohort was restricted to cases with pT1b-region disease (pT1b, pT1b1, pT1b2, or pT1b3) and complete availability of the prespecified 20-feature immunohistochemical immune panel, yielding 169 patients for the primary complete-case clustering analysis.

The study was approved by the Ethics Committee of Medical University Pleven (protocol code 656, approved on 29 June 2021). All procedures were performed in accordance with the Declaration of Helsinki. Written informed consent was obtained from all participants.

### Histology and clinicopathological evaluation

Histological diagnosis was established on hematoxylin and eosin-stained whole tissue sections by experienced pathologists according to routine diagnostic criteria and procedures described in our previous publications (17–20). Tumors were categorized as squamous cell carcinoma or non-squamous carcinoma for statistical modeling; the latter included adenocarcinoma and adenosquamous carcinoma. Tumor size, pT category, nodal status, FIGO stage, grade, and lymphovascular space invasion (LVSI) were extracted from pathology and/or local clinical records. Because pT1b subclasses are size-based, tumor size and pT subclass were not interpreted as independent variables in the main clinicopathological summary. Representative tissue blocks were selected after review of diagnostic slides to include viable invasive carcinoma and tumor-associated stroma when present. Areas of necrosis, ulceration, cautery artifact, and non-representative inflammation unrelated to invasive tumor were avoided where possible.

The staging system reflected the locally recorded FIGO and pathological categories available in the study database. Treatment and HPV-status variables were not present in the analytic dataset and therefore could not be incorporated into adjustment or sensitivity models.

### Immunohistochemistry and scoring

Immunohistochemical staining was performed on formalin-fixed paraffin-embedded tissue sections using a polymer-based detection workflow consistent with our previous reports (17–20). Antibody-defined markers, derived immune features, and scoring schemes are summarized in the **Supplementary Material**. Immune-cell markers were scored separately in the intratumoral (IT) compartment, defined as immune cells within tumor nests or in direct contact with malignant epithelium, and in the stromal (ST) compartment, defined as immune cells within tumor-associated stroma. PD-L1 and CD47 were scored as tumor or immunomodulatory markers according to the study scoring scheme.

Histologic and immunohistochemical assessments were performed before the present unsupervised clustering and survival analyses; evaluators were not provided with the final cluster assignments. Immunohistochemical scoring was performed by two evaluating pathologists. When discrepancies in evaluation occurred, the slides and scores were reviewed jointly and a final consensus score was agreed between the two pathologists before entry into the analytic dataset. Because only consensus scores were retained, paired observer-level values were not available for retrospective calculation of weighted kappa or intraclass correlation coefficients.

### Immunohistochemical feature panel and clustering

The primary panel comprised 20 immunohistochemical immune features rather than 20 independent antibodies. These features were PD-L1, CD47 H-score, CD8-IT, CD8-ST, CD68-IT, CD68-ST, PD-1-IT, PD-1-ST, CTLA-4-IT, CTLA-4-ST, CD20-IT, CD20-ST, CD3-IT, CD3-ST, CD57-IT, CD57-ST, CD4-IT, CD4-ST, FOXP3-IT, and FOXP3-ST. Feature values were standardized before clustering. Hierarchical clustering used Euclidean distance and Ward.D2 linkage. The three-cluster solution was retained as the primary unsupervised result because it provided biologically interpretable phenotypes and continuity with the planned analysis of coordinated immune states. Silhouette width, gap statistic, bootstrap Jaccard stability, and alternative clustering sensitivity analyses were used to evaluate robustness but were not used as independent proof of subtype discreteness.

### Statistical analysis

Continuous variables were summarized as median and interquartile range and compared using rank-based tests. Categorical variables were summarized as counts and percentages and compared using Fisher exact or chi-square tests as appropriate. **Table 1** reports both global three-cluster p values and binary p values comparing C2 with combined C1/C3. The latter contrast was exploratory and outcome-informed, generated after inspection of the primary three-cluster survival analysis; the original three-cluster solution remained the primary unsupervised analysis.

**Table 1:** Clinicopathological characteristics by tumor immune microenvironment cluster.

Characteristic	Level	Overall	C1	C2	C3	Global p value	C2 vs C1/C3 p value
Age, years	Median (IQR)	49.0 (43.0-60.0)	45.5 (39.0-55.0)	51.0 (45.0-61.0)	50.0 (43.0-60.0)	0.022	0.036
Histology	Squamous cell carcinoma	116 (68.6)	36 (72.0)	47 (60.3)	33 (80.5)	0.073	0.032
	Non-squamous carcinoma	53 (31.4)	14 (28.0)	31 (39.7)	8 (19.5)		
N status	N0	131 (77.5)	40 (80.0)	60 (76.9)	31 (75.6)	0.887	1.000
	N1	38 (22.5)	10 (20.0)	18 (23.1)	10 (24.4)		
Tumor size	<2 cm	65 (38.5)	17 (34.0)	32 (41.0)	16 (39.0)	0.709	0.873
	2–4 cm	79 (46.7)	27 (54.0)	35 (44.9)	17 (41.5)		
	>4 cm	25 (14.8)	6 (12.0)	11 (14.1)	8 (19.5)		
FIGO stage	FIGO I	131 (77.5)	40 (80.0)	60 (76.9)	31 (75.6)	0.892	1.000
	FIGO III	38 (22.5)	10 (20.0)	18 (23.1)	10 (24.4)		
Grade	G1	47 (27.8)	14 (28.0)	22 (28.2)	11 (26.8)	0.428	0.278
	G2	78 (46.2)	23 (46.0)	40 (51.3)	15 (36.6)		
	G3	44 (26.0)	13 (26.0)	16 (20.5)	15 (36.6)		
LVSI	No	137 (81.1)	39 (78.0)	68 (87.2)	30 (73.2)	0.127	0.076
	Yes	32 (18.9)	11 (22.0)	10 (12.8)	11 (26.8)		

Global p values compare C1, C2, and C3. Binary p values compare the exploratory C2 versus combined C1/C3 contrast. Values are median (IQR) or n (%). p values are unadjusted exploratory comparisons.

pT1b subclasses are size-based and are therefore not shown separately from tumor size in the main table.

Between-cluster feature differences were assessed using Kruskal-Wallis tests with Benjamini-Hochberg false-discovery-rate (FDR) adjustment and epsilon-squared effect sizes. Because the same features were used to construct the clusters, these tests were interpreted descriptively as feature-discrimination summaries rather than independent confirmatory tests.

Overall survival was assessed using Kaplan-Meier curves, log-rank tests, and Cox regression. Neoplasm-specific death was analyzed using cumulative-incidence functions, Fine-Gray regression, and cause-specific Cox regression sensitivity analyses; deaths from other causes were treated as competing events. Cause of death was coded from available clinical follow-up information and grouped as neoplasm-specific or other-cause death. Unknown or non-neoplastic fatal events were not counted as neoplasm-specific deaths. Multivariable models adjusted for age, nodal status, and histology, selected *a priori* as core clinical variables with a manageable number of covariates given the event count. Sensitivity models additionally considered LVSI, tumor size, grade, pT category, and FIGO stage when estimable. Proportional-hazards assumptions were tested using Schoenfeld residuals. Penalized multinomial regression with least absolute shrinkage and selection operator (LASSO) was used only as internal cluster-reproduction quality control because cluster labels were derived from the same features used as predictors.

## Results

The final complete-case clustering cohort included 169 patients from the source dataset after restriction to pT1b-region disease and complete 20-feature panel availability. Median age was 49 years (IQR 43-60), and median follow-up was 7.0 years (IQR 4.2-9.9). The analysis should therefore be interpreted as a complete-case exploratory analysis of surgically treated pT1b-region cervical cancer.

The primary three-cluster solution included C1 (n=50, 29.6%), C2 (n=78, 46.2%), and C3 (n=41, 24.3%). Clinicopathological distributions are shown in **Table 1**. C2 patients were older than the combined C1/C3 group (binary p=0.036) and had a higher proportion of non-squamous histology (39.7% in C2 vs 23.1% in C1/C3; binary p=0.032). Nodal status, tumor size, FIGO stage, grade, and LVSI were not materially different in the central C2 versus C1/C3 comparison. After FDR correction across exploratory clinicopathological tests, these associations did not remain statistically robust, supporting cautious interpretation.

**Figure 1:**
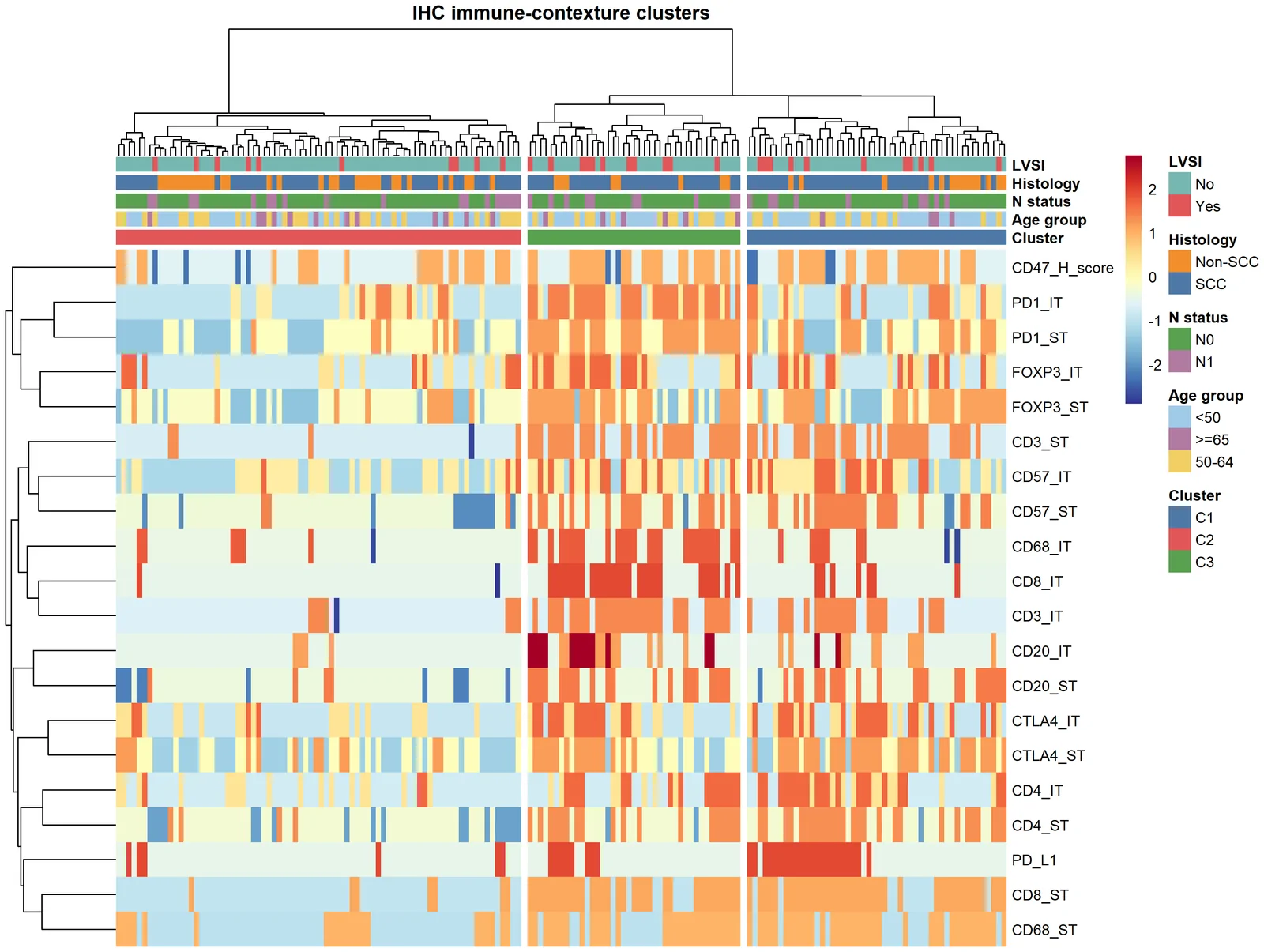
Tumor immune microenvironment heatmap. Unsupervised hierarchical clustering of the 20-feature immunohistochemical immune panel identified three exploratory clusters. Columns represent patients and rows represent immune features. Top annotations show cluster, age group, nodal status, histology, and LVSI. IT denotes intratumoral and ST denotes stromal scoring.

The clusters showed distinct but partially overlapping immune-feature profiles (**Figure 1**). All 20 immune features differed across the three clusters after FDR correction; because the features defined the clusters, these results are best interpreted as descriptive summaries of cluster-defining structure. The strongest discriminators were T-cell and compartment-specific features: CD8-ST, CD8-IT, CD3-ST, PD-1-ST, PD-1-IT, CD3-IT, CD4-ST, CD68-IT, CD20-ST, and CD57-ST (**Table 2**). C2 was characterized by relatively low stromal and intratumoral lymphoid and checkpoint-associated scores. C3 represented the most broadly immune-inflamed phenotype, whereas C1 showed a mixed or stromal-weighted profile.

**Table 2:** Top discriminatory immunohistochemical immune features defining the three clusters.

Feature	C1 mean	C2 mean	C3 mean	Epsilon-squared	FDR p
CD8-ST	1.78	1.09	1.78	0.47	<0.001
CD8-IT	1.14	1.00	1.63	0.38	<0.001
CD3-ST	1.58	1.04	1.71	0.37	<0.001
PD-1-ST	1.16	0.69	1.73	0.30	<0.001
PD-1-IT	0.70	0.41	1.61	0.30	<0.001
CD3-IT	1.42	1.08	1.71	0.28	<0.001
CD4-ST	1.52	0.82	1.39	0.27	<0.001
CD68-IT	1.10	1.06	1.61	0.26	<0.001
CD20-ST	1.40	0.91	1.49	0.23	<0.001
CD57-ST	1.42	0.88	1.44	0.23	<0.001

Features are ranked by FDR-adjusted p value from Kruskal-Wallis tests. Because these features were used to construct the clusters, the table is descriptive rather than confirmatory. IT, intratumoral; ST, stromal.

Cluster-number assessment supported a cautious interpretation. Mean silhouette width was highest for k=2 (0.169), while the k=3 solution had a lower but still interpretable mean silhouette width (0.144). Gap statistic values were similar for k=2 and k=3 (0.514 and 0.507, respectively). Bootstrap Jaccard stability was strongest for C2 (mean Jaccard 0.795), whereas C1 and C3 were less stable (0.406 and 0.499). Alternative clustering specifications produced adjusted Rand indices ranging from 0.242 to 0.488 relative to the primary solution. These findings support the reproducibility of the immune-depleted C2 tendency more strongly than the discreteness of all three clusters.

**Figure 2:**
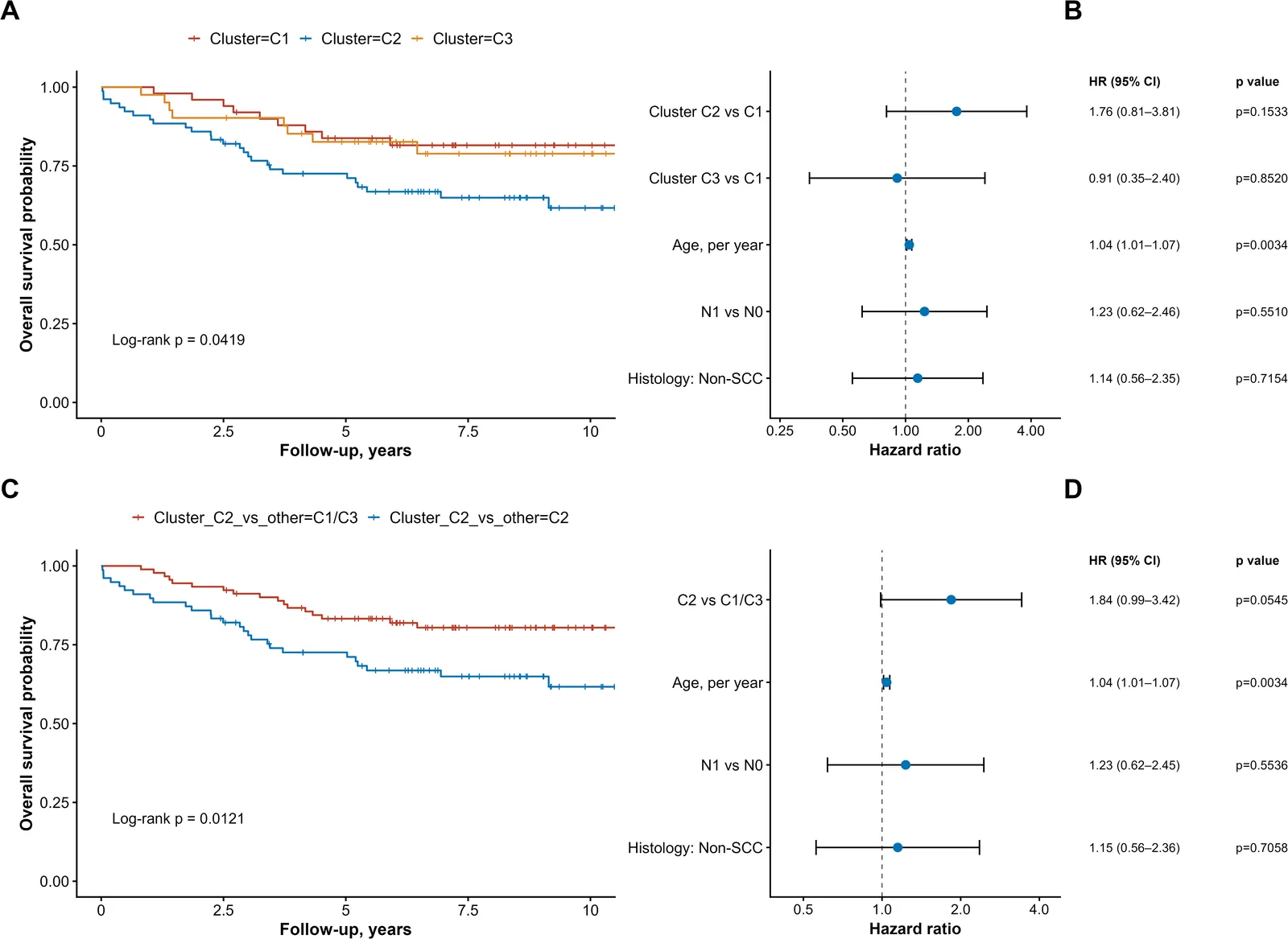
Overall survival according to tumor immune microenvironment clusters. **(A)** Kaplan-Meier curves for C1, C2, and C3. **(B)** multivariable Cox model for the three-cluster solution adjusted for age, nodal status, and histology. **(C)** Kaplan-Meier curves for the exploratory C2 versus combined C1/C3 contrast. **(D)** multivariable Cox model for C2 versus C1/C3 adjusted for age, nodal status, and histology. HR, hazard ratio; CI, confidence interval.

Overall survival differed among the three primary clusters (log-rank p=0.042). In adjusted Cox regression, C2 showed an adverse but non-significant association compared with C1 (HR 1.76, 95% CI 0.81-3.81, p=0.153), while C3 did not differ from C1 (HR 0.91, 95% CI 0.35-2.40, p=0.852). In the exploratory C2 versus C1/C3 contrast, C2 was associated with inferior overall survival by Kaplan-Meier analysis (log-rank p=0.012) and univariable Cox regression (HR 2.14, 95% CI 1.16-3.92, p=0.014). After adjustment for age, nodal status, and histology, the estimate remained directionally adverse but borderline (HR 1.84, 95% CI 0.99-3.42, p=0.055) (**Figure 2**; **Supplementary Table 7**). Proportional-hazards testing did not suggest violation for the core C2 model (global p=0.639; **Supplementary Table 10**).

Sensitivity analyses showed a generally consistent adverse direction for C2, but with limited precision. The C2 estimate was similar after adjustment for tumor size (HR 1.83, 95% CI 0.99-3.41, p=0.055), pT category (HR 1.79, 95% CI 0.96-3.35, p=0.066), and FIGO stage instead of nodal status (HR 1.84, 95% CI 0.99-3.42, p=0.055). Models additionally adjusted for LVSI or grade yielded stronger C2 estimates, but these were considered exploratory because of reduced sample size, model instability, and event limitation.

**Figure 3:**
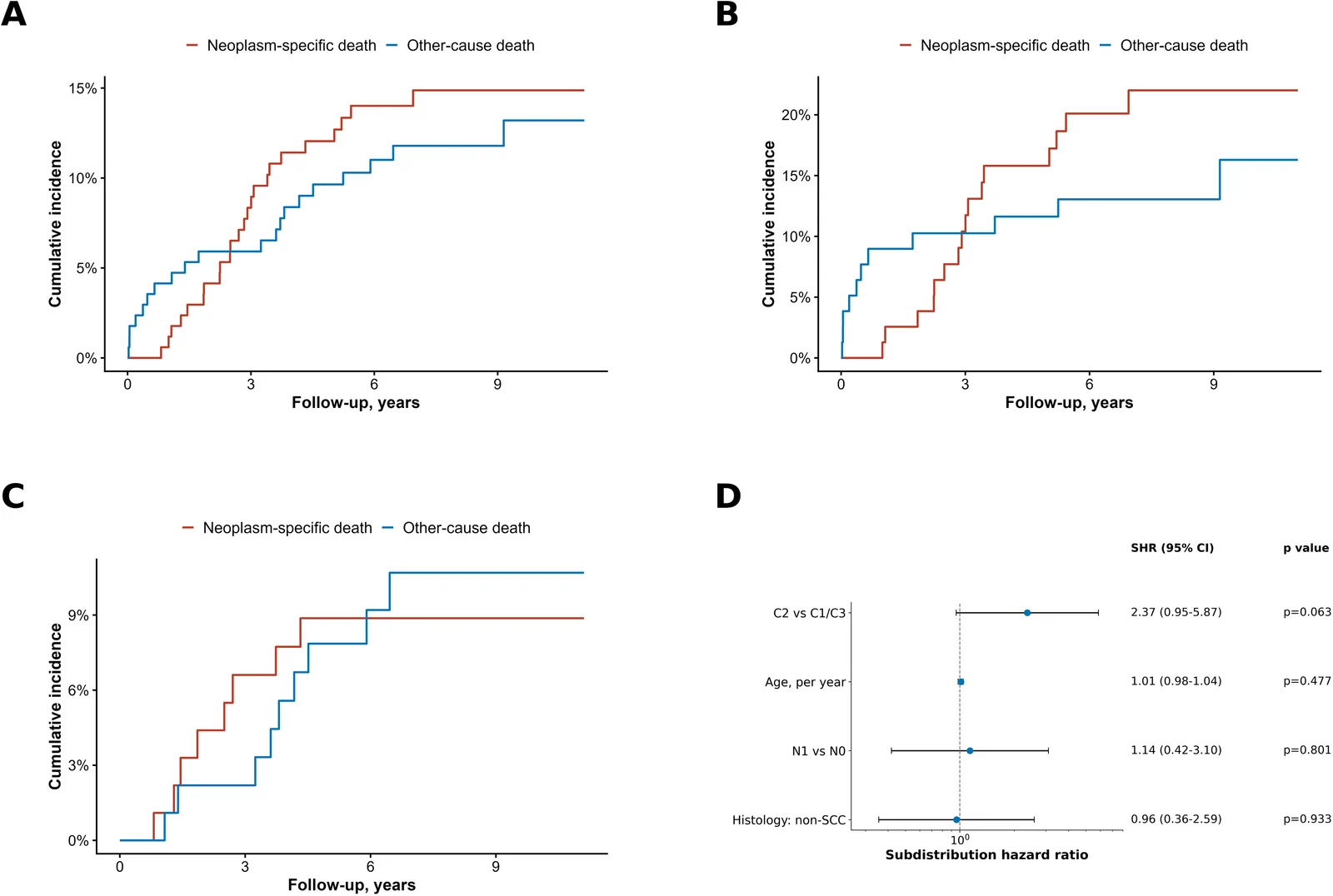
Competing-risk analysis. **(A)**, cumulative incidence of neoplasm-specific and other-cause death in the entire cohort. **(B)** cumulative incidence within the C2 subgroup. **(C)** cumulative incidence within the combined C1/C3 subgroup. **(D)** multivariable Fine-Gray model for neoplasm-specific death comparing C2 with C1/C3, adjusted for age, nodal status, and histology. SHR, subdistribution hazard ratio; CI, confidence interval.

There were 44 overall deaths, including 24 neoplasm-specific deaths and 20 other-cause deaths. C2 included 16 neoplasm-specific deaths, compared with 3 in C1 and 5 in C3. In the three-cluster Fine-Gray model adjusted for age, nodal status, and histology, C2 showed the strongest adverse direction compared with C1 (SHR 3.49, 95% CI 0.97-12.59, p=0.056). In the exploratory binary Fine-Gray model, C2 again showed a borderline adverse direction compared with C1/C3 (SHR 2.37, 95% CI 0.95-5.87, p=0.063). Cause-specific Cox regression supported the same direction and was statistically significant for C2 versus C1/C3 (HR 2.52, 95% CI 1.06-6.01, p=0.037), although this sensitivity analysis was based on only 24 neoplasm-specific deaths. Competing-risk curves are shown in **Figure 3**, with supporting models in **Supplementary Tables 8**, **9**.

LASSO multinomial regression was retained as an internal descriptive quality-control analysis. At lambda.min, cross-validated classification error was 0.095 and the adjusted Rand index relative to the original clusters was 1.000; at lambda.1se, cross-validated error was 0.112 and the adjusted Rand index was 0.940. Because the cluster labels were derived from the same immune-feature matrix used as predictors, this analysis does not provide independent validation and should not be interpreted as a reduced diagnostic panel (**Supplementary Figure 5**; **Supplementary Table 12**).

## Discussion

### Summary of main findings

This exploratory study identified three tumor immune microenvironment phenotypes in cervical cancer using a 20-feature, compartment-aware immunohistochemical panel. The most consistent biological finding was the emergence of C2 as a relatively immune-depleted cluster, defined by lower coordinated lymphoid and checkpoint-associated features. The survival and competing-risk analyses pointed in an unfavorable direction for C2, but the adjusted estimates were borderline and should be interpreted as hypothesis-generating rather than as proof of an independently adverse prognostic classifier.

### Interpretation in context

The strongest between-cluster feature differences were dominated by CD8, CD3, PD-1, CD4, CD68, CD20, and CD57 features, with several of the largest effects observed in the stromal compartment. This supports the view that immune architecture in cervical cancer cannot be adequately summarized by a single marker or by a simple high-versus-low lymphocyte score. The importance of T-cell infiltration, particularly CD8-positive cells, is consistent with systematic and clinical literature on cervical carcinogenesis and invasive cervical cancer (6, 7, 21).

The C2 cluster showed simultaneous relative depletion of stromal CD8 and CD3, intratumoral CD8 and CD3, PD-1-positive cells, CD20-positive cells, CD57-positive cells, and several regulatory or checkpoint-related features. This pattern may reflect reduced antigen-experienced lymphoid infiltration, impaired immune recruitment, or a tumor microenvironment that is less visibly engaged by adaptive immune responses. Conversely, C3 combined high intratumoral and stromal lymphoid features with PD-1 and FOXP3 signals, suggesting an immune-inflamed but immunoregulatory phenotype. PD-1 and PD-L1 biology in cervical cancer is heterogeneous, and prior work suggests that checkpoint expression may be linked to interferon-driven immune infiltration rather than being directly interpretable as a stand-alone marker of immune escape (8–10, 28).

Macrophage- and B-cell-related features also contributed to cluster definition. CD68-IT was among the top discriminatory variables, and CD20-ST showed a strong between-cluster difference. Tumor-associated macrophages have been associated with cervical cancer progression and immune modulation, particularly when macrophage polarization and stromal organization are considered (11, 12). B-cell infiltration and tertiary lymphoid structures are increasingly recognized as relevant to anti-tumor immunity and outcome in HPV-associated and other solid tumors (13, 22). In this study, these features were not used to claim isolated prognostic biomarkers; rather, they helped define a broader coordinated immune phenotype.

The present analysis extends our previous cervical cancer studies, which examined CD47, CD8/CD68, phagocytic and immunomodulatory markers, and CD4/FOXP3 patterns in overlapping Bulgarian cohorts (17–20). The novelty of the current work is not the first evaluation of these antibodies, but the unsupervised integration of compartment-specific features into a coordinated immune-profile framework, the explicit assessment of cluster robustness, and the use of competing-risk and sensitivity analyses.

The findings also need to be viewed in the context of emerging high-resolution approaches. Single-cell and spatial studies of cervical cancer have mapped exhausted T-cell states, tumor-specific germinal-center B cells, immune-excluded stromal landscapes, and microenvironmental programs linked to prognosis (14–16). Spatial transcriptomics and multiplex imaging can capture cell-cell neighborhoods with much greater resolution than conventional immunohistochemistry (23–27). However, these platforms require specialized tissue processing, instrumentation, computational pipelines, and expertise that are not yet available in many routine pathology settings. A compartment-aware immunohistochemical feature panel may therefore be a pragmatic intermediate approach, but it should not be presented as a substitute for spatial technologies or as a validated clinical decision tool.

The broader immunotherapy biomarker literature in gynecologic oncology emphasizes that immune-related markers are difficult to translate into clinically meaningful predictors without rigorous validation. Recent reviews of predictive biomarkers for immune checkpoint inhibition in gynecologic malignancies highlight the need to distinguish prognostic associations, predictive biomarkers, and practical pathology assays (29). This distinction is central to the present study: the C2 phenotype is an exploratory immune-depleted pattern with unfavorable survival signals, not a validated predictor of treatment benefit.

### Strengths and limitations

Strengths include the use of routine immunohistochemistry, separate IT/ST assessment, consensus scoring by two evaluating pathologists when discrepancies occurred, long follow-up, cause-of-death coding, competing-risk analysis, and additional sensitivity analyses.

Limitations are substantial. The study is retrospective and single-cohort, and no independent validation cohort was available. The C2 versus C1/C3 contrast was exploratory and outcome-informed, which may inflate the apparent strength of the survival association. The adjusted Cox and Fine-Gray estimates were borderline. The three-cluster structure had modest silhouette values and only C2 showed comparatively stronger bootstrap stability. The IHC scores were semi-quantitative and ordinal, making the Euclidean/Ward.D2 clustering a pragmatic but imperfect choice. Alternative distance and linkage choices did not fully reproduce the primary clustering structure. Treatment and HPV-status variables were unavailable. Although discrepancies were resolved by consensus between two evaluating pathologists, formal interobserver reproducibility coefficients could not be calculated because only final consensus scores were retained. Finally, the number of deaths, especially neoplasm-specific deaths, limited the precision of multivariable models and increased the risk of overfitting.

### Conclusions

Unsupervised analysis of a 20-feature, compartment-aware immunohistochemical immune panel identified exploratory cervical cancer tumor immune microenvironment phenotypes. The C2 cluster showed relative immune depletion and unfavorable survival signals, but the adjusted associations were borderline and require independent validation. The results support further study of practical, routine immunohistochemical immune profiling as a bridge between single-marker pathology and higher-dimensional spatial approaches.

## Data Availability

The original contributions presented in the study are included in the article/Supplementary Material. Further inquiries can be directed to the corresponding authors.
