# Supplementary material for: Unsupervised 20-feature immunohistochemical immune profiling identifies exploratory cervical cancer phenotypes with unfavorable survival signals
Source: Front Oncol. 2026 Aug 24;16:1914836. doi: 10.3389/fonc.2026.1914836 (PMC13547501; doi:10.3389/fonc.2026.1914836)
Supplement: Supplementary file 1 [file DataSheet1.pdf]

Supplementary material

Unsupervised 20-feature immunohistochemical immune profiling identifies exploratory cervical cancer phenotypes with unfavorable survival signals

Supplementary Table S1. Antibody-defined markers and derived immunohistochemical immune features. IT, intratumoral; ST, stromal.

Antibody marker	Features generated	Biological role	Compartment evaluated	Scoring
CD3	CD3-IT; CD3-ST	Pan-T lymphocytes	IT and ST	Semi-quantitative 0-2
CD4	CD4-IT; CD4-ST	Helper T-cell enriched lymphocytes	IT and ST	Semi-quantitative 0-2
CD8	CD8-IT; CD8-ST	Cytotoxic T-cell enriched lymphocytes	IT and ST	Semi-quantitative 0-2
CD20	CD20-IT; CD20-ST	B lymphocytes	IT and ST	Semi-quantitative 0-2
CD57	CD57-IT; CD57-ST	NK/T-cell associated marker	IT and ST	Semi-quantitative 0-2
CD68	CD68-IT; CD68-ST	Macrophage-associated marker	IT and ST	Semi-quantitative 0-2
FOXP3	FOXP3-IT; FOXP3-ST	Regulatory T-cell associated marker	IT and ST	Semi-quantitative 0-2
PD-1	PD-1-IT; PD-1-ST	Checkpoint/exhaustion-associated receptor	IT and ST	Semi-quantitative 0-2
CTLA-4	CTLA-4-IT; CTLA-4-ST	Checkpoint/regulatory receptor	IT and ST	Semi-quantitative 0-2
PD-L1	PD-L1	Checkpoint ligand	Tumor/immune microenvironment	Study scoring scheme
CD47	CD47 H-score	Anti-phagocytic checkpoint	Tumor/immunomodulatory marker	H-score

Note: The primary analytic panel comprised 20 derived immunohistochemical immune features or scores, not 20 independent antibodies. Immunohistochemical scoring was performed by two evaluating pathologists. When discrepancies occurred, slides and scores were reviewed jointly and a final consensus score was agreed before inclusion in the analytic dataset.

Supplementary Table S2. Missingness of the 20-feature immunohistochemical immune panel among pT1b-region eligible cases.

feature	missing_n	eligible_n	missing_percent
PD_L1	3	177	1.7
CD47_H_score	2	177	1.1
CD8_IT	3	177	1.7
CD8_ST	3	177	1.7
CD68_IT	2	177	1.1
CD68_ST	2	177	1.1
PD1_IT	3	177	1.7
PD1_ST	2	177	1.1
CTLA4_IT	4	177	2.3
CTLA4_ST	4	177	2.3
CD20_IT	4	177	2.3
CD20_ST	4	177	2.3
CD3_IT	3	177	1.7
CD3_ST	3	177	1.7
CD57_IT	3	177	1.7
CD57_ST	3	177	1.7
CD4_IT	3	177	1.7
CD4_ST	3	177	1.7
FOXP3_IT	4	177	2.3
FOXP3_ST	4	177	2.3

Supplementary Table S3. Silhouette statistics for k=2-8.

k	Mean silhouette width	Minimum silhouette width
2	0.169	-0.205
3	0.144	-0.169
4	0.117	-0.147
5	0.121	-0.117
6	0.107	-0.152
7	0.109	-0.152
8	0.053	-0.152

Supplementary Table S4. Gap statistic for candidate cluster numbers.

k	Gap statistic	Simulation SE
1	0.459	0.009
2	0.514	0.010
3	0.507	0.009

4	0.509	0.010
5	0.511	0.010
6	0.510	0.009
7	0.508	0.009
8	0.512	0.009

Supplementary Table S5. Bootstrap cluster stability. C2 showed the strongest stability.

Cluster	Mean Jaccard	Dissolution count	Recovery count	Mean feature burden
C1	0.406	406	4	1.186
C2	0.795	2	360	0.739
C3	0.499	276	20	1.345

Supplementary Table S6. Sensitivity of clustering to alternative distance, scaling, and linkage choices.

Alternative clustering specification	Adjusted Rand index
Euclidean_WardD2_scaled_primary	1.000
Manhattan_WardD2_scaled	0.488
Manhattan_WardD2_unscaled_ordinal	0.436
Manhattan_PAM_scaled	0.434
Gower_PAM_scaled	0.393
Euclidean_WardD2_unscaled_ordinal	0.366
Manhattan_average_scaled	0.268
Euclidean_average_scaled	0.242

Supplementary Table S7. Overall survival Cox models for C2-related terms across core and sensitivity models.

Model	n	Events	Cluster term	HR (95% CI)	p value
OS_core_three_cluster_age_N_histology	169	44	ClusterC2	1.76 (0.81-3.81)	0.153
OS_core_C2_vs_C1C3_age_N_histology	169	44	Cluster_C2_vs_C1C3C2	1.84 (0.99-3.42)	0.055
OS_sensitivity_plus_LVSI	154	37	Cluster_C2_vs_C1C3C2	2.06 (1.05-4.03)	0.035
OS_sensitivity_plus_tumor_size	169	44	Cluster_C2_vs_C1C3C2	1.83 (0.99-3.41)	0.055
OS_sensitivity_plus_grade	169	44	Cluster_C2_vs_C1C3C2	2.08 (1.11-3.91)	0.023
OS_sensitivity_age_FIGO_histology	169	44	Cluster_C2_vs_C1C3C2	1.84 (0.99-3.42)	0.055
OS_sensitivity_age_pT_histology	169	44	Cluster_C2_vs_C1C3C2	1.79 (0.96-3.35)	0.066

Supplementary Table S8. Fine-Gray competing-risk models for C2-related terms across core and sensitivity models.

Model	n	Neoplasm-specific deaths	Other-cause deaths	Cluster term	SHR (95% CI)	p value
FG_three_cluster_age_N_histology	169	24	20	ClusterC2	3.49 (0.97-12.59)	0.056
FG_C2_vs_C1C3_age_N_histology	169	24	20	Cluster_C2_vs_C1C3C2	2.37 (0.95-5.87)	0.063
FG_sensitivity_plus_LVSI	154	19	18	Cluster_C2_vs_C1C3C2	3.11 (1.11-8.71)	0.031
FG_sensitivity_plus_tumor_size	169	24	20	Cluster_C2_vs_C1C3C2	2.40 (0.96-5.95)	0.060
FG_sensitivity_age_FIGO_histology	169	24	20	Cluster_C2_vs_C1C3C2	2.37 (0.95-5.87)	0.063

Supplementary Table S9. Cause-specific Cox sensitivity models for neoplasm-specific death.

Model	n	Neoplasm-specific deaths	Cluster term	HR (95% CI)	p value
CS_Cox_three_cluster_age_N_histology	169	24	ClusterC2	3.62 (1.03-12.74)	0.045
CS_Cox_C2_vs_C1C3_age_N_histology	169	24	Cluster_C2_vs_C1C3C2	2.52 (1.06-6.01)	0.037
CS_Cox_sensitivity_plus_LVSI	154	19	Cluster_C2_vs_C1C3C2	3.33 (1.24-8.97)	0.017
CS_Cox_sensitivity_plus_tumor_size	169	24	Cluster_C2_vs_C1C3C2	2.47 (1.04-5.87)	0.040
CS_Cox_sensitivity_age_FIGO_histology	169	24	Cluster_C2_vs_C1C3C2	2.52 (1.06-6.01)	0.037

Supplementary Table S10. Global proportional-hazards assumption tests for Cox models.

Model	Global PH p value
CS_Cox_C2_vs_C1C3_age_N_histology	0.341
CS_Cox_sensitivity_age_FIGO_histology	0.341

CS_Cox_sensitivity_plus_LVSI	0.959
CS_Cox_sensitivity_plus_tumor_size	0.177
CS_Cox_three_cluster_age_N_histology	0.459
OS_core_C2_vs_C1C3_age_N_histology	0.639
OS_core_three_cluster_age_N_histology	0.746
OS_sensitivity_age_FIGO_histology	0.639
OS_sensitivity_age_pT_histology	0.861
OS_sensitivity_plus_grade	0.758
OS_sensitivity_plus_LVSI	0.921
OS_sensitivity_plus_tumor_size	0.862

Supplementary Table S11. Availability of treatment and HPV variables in the analysis dataset.

requested_variable	detected_columns	note
Adjuvant treatment / radiotherapy / chemoradiotherapy	Not detected	No treatment-related column detected in input dataset.
HPV status / HPV genotype	Not detected	No HPV-related column detected in input dataset.

Note: Treatment and HPV-status variables were not present in the analytic dataset and could not be incorporated into adjustment or sensitivity models.

Supplementary Table S12. LASSO internal cluster-reproduction summary.

Penalty level	Cross-validated classification error	Adjusted Rand index	Interpretation
lambda.min	0.095	1.000	Internal reconstruction of original clusters; not independent validation.
lambda.1se	0.112	0.940	More regularized internal reconstruction; not a reduced clinical panel.

Supplementary Table S13. Immunohistochemical scoring reproducibility note.

Topic	Available information	Implication for current analysis
Scoring procedure	IHC scores were evaluated by two pathologists. Discrepancies were resolved by joint review and consensus before data entry.	The analytic dataset contains final consensus scores.
Interobserver coefficients	Paired observer-level scores were not retained in the analytic dataset.	Weighted kappa or intraclass correlation coefficients could not be calculated retrospectively.
Future reporting	Independent observer-level scoring would allow weighted kappa for ordinal scores and ICC for continuous/H-score variables.	External validation should include formal reproducibility reporting.

Supplementary Figure S1. Cluster-number assessment by mean silhouette width. Mean silhouette width was calculated for k=2-8. The k=3 solution was retained as the primary biologically interpretable unsupervised solution, while the modest values support interpretation as partially overlapping phenotypes rather than sharply discrete subtypes.

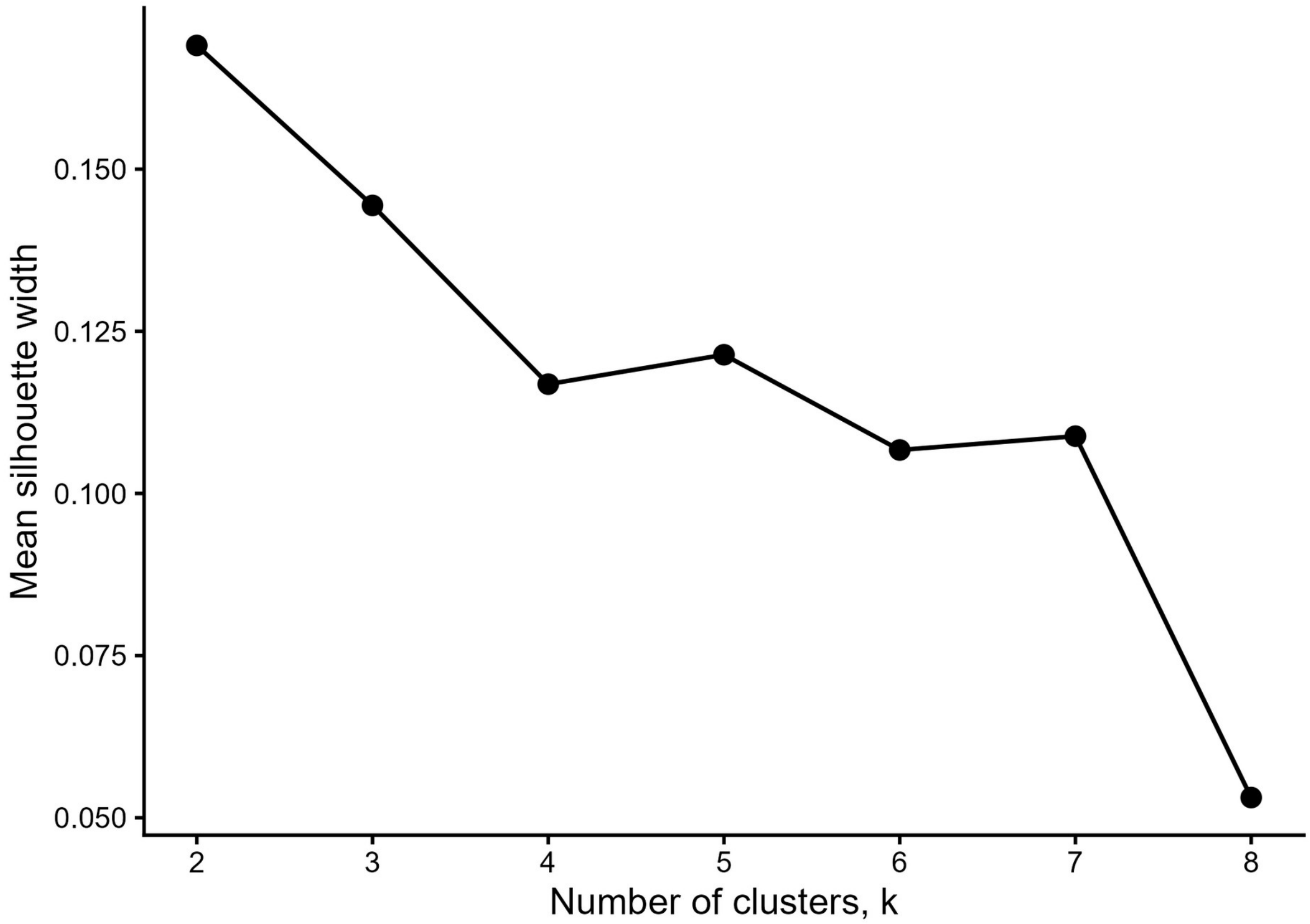

Supplementary Figure S2. Gap-statistic assessment across candidate cluster numbers.

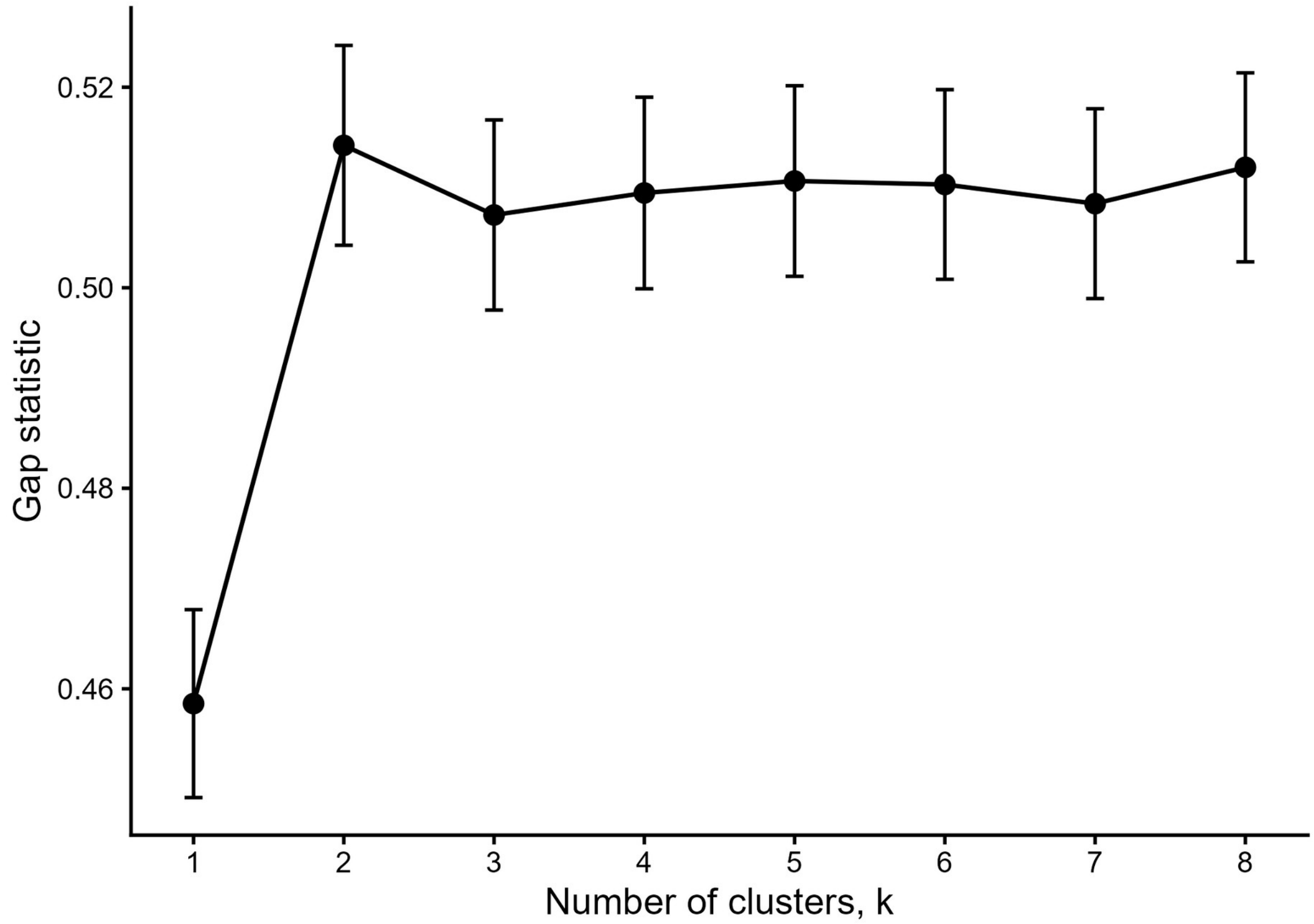

Supplementary Figure S3. Principal component analysis of the scaled 20-feature immunohistochemical immune profile. Each point represents one tumor sample and is colored/shaped according to the three-cluster solution.

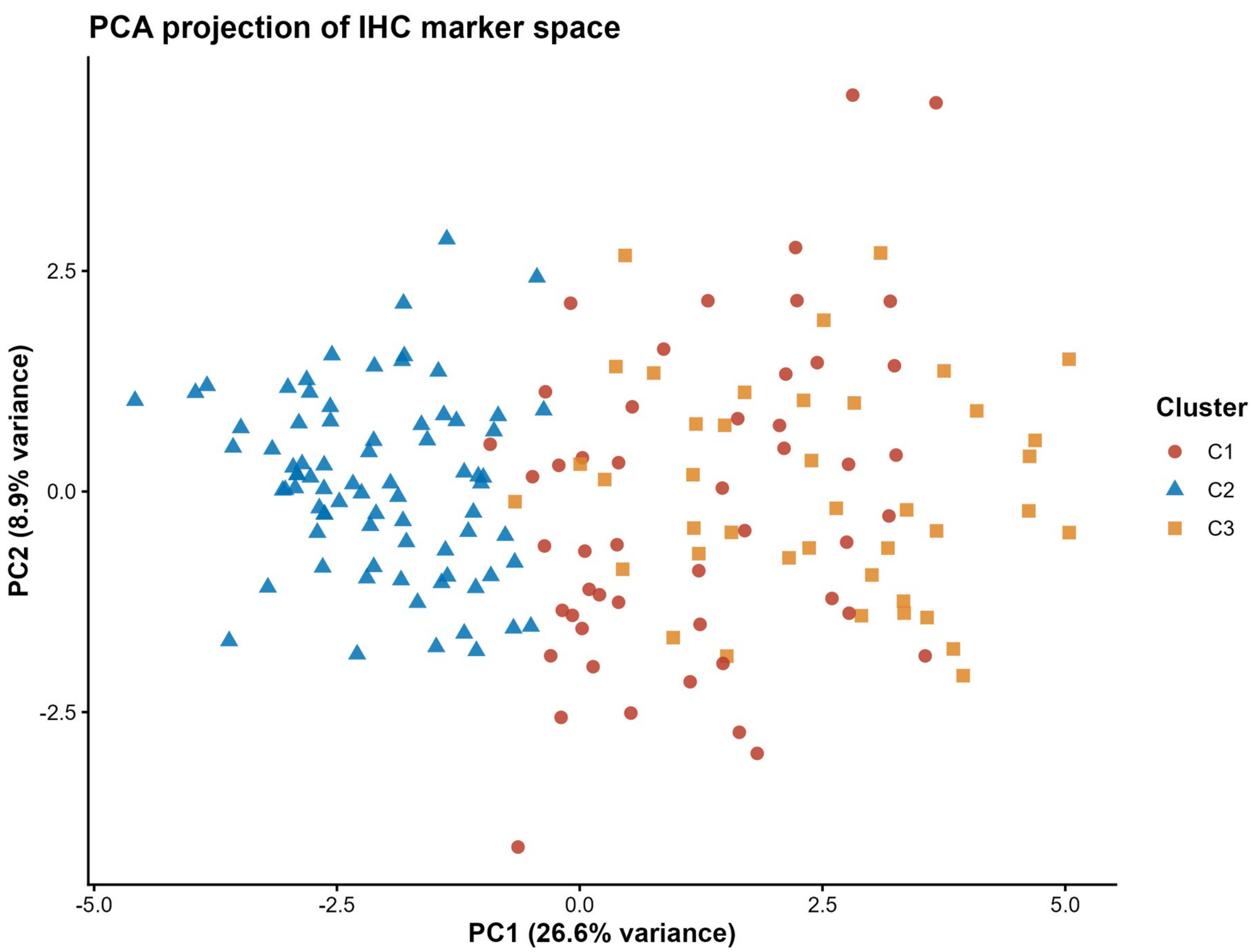

Supplementary Figure S4. Median immune-feature scores across the three clusters. Scores are shown on the original semi-quantitative scale from 0 to 2.

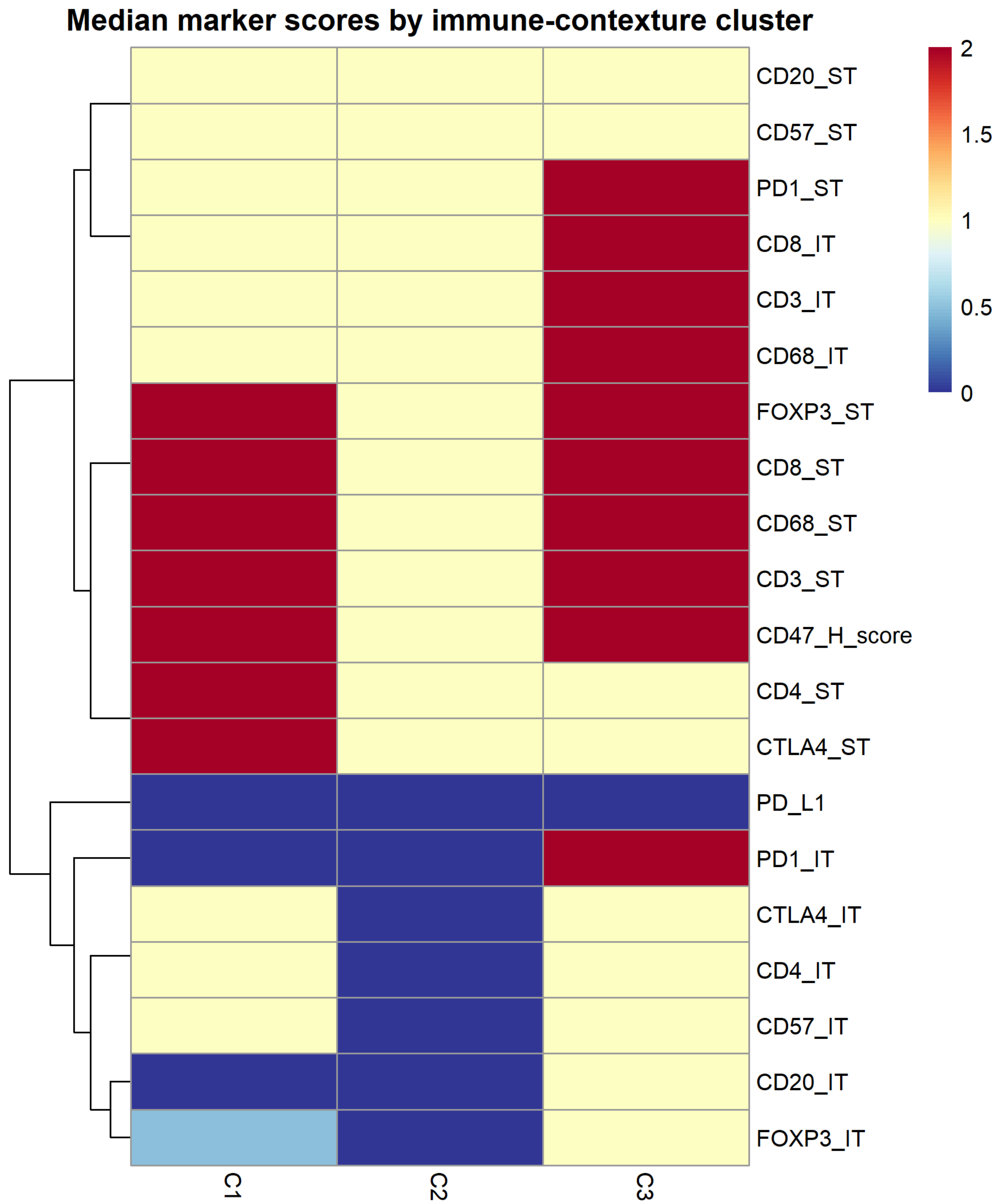

Supplementary Figure S5. LASSO-based internal cluster-reproduction quality control. Because target cluster labels were derived from the same features used as predictors, this analysis should not be interpreted as independent validation or reduced-panel development.

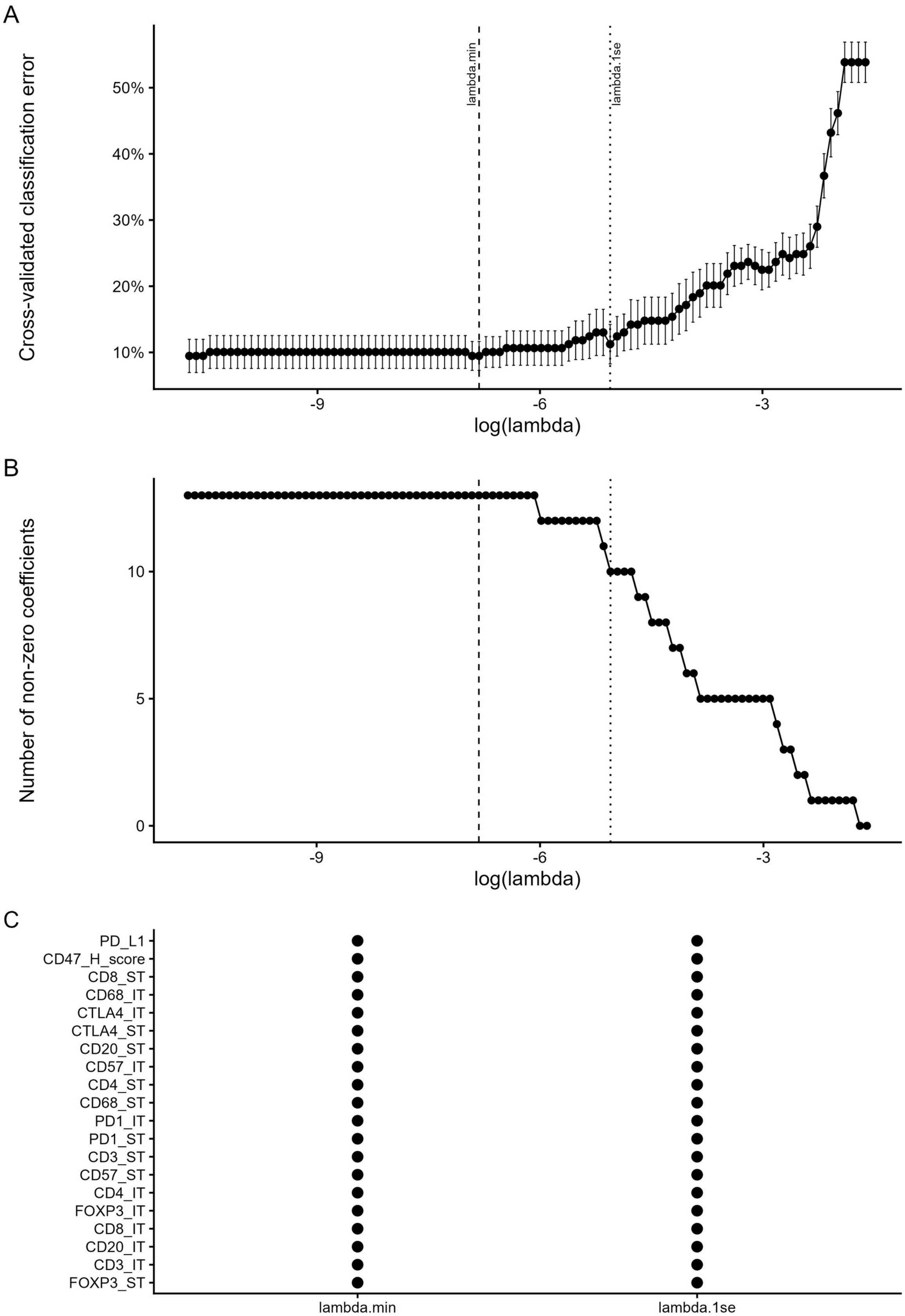
A
Cross-validated classification error
50%
40%
30%
20%
10%
lambda.min
lambda.1se
-9
-6
-3
log(lambda)
B
Number of non-zero coefficients
10
5
0
-9
-6
-3
log(lambda)
C
PD_L1
CD47_H_score
CD8_ST
CD68_IT
CTLA4_IT
CTLA4_ST
CD20_ST
CD57_IT
CD4_ST
CD68_ST
PD1_IT
PD1_ST
CD3_ST
CD57_ST
CD4_IT
FOXP3_IT
CD8_IT
CD20_IT
CD3_IT
FOXP3_ST
lambda.min
lambda.1se
